# Employment quality and workers’ health and well-being in China: longitudinal evidence from the China Family Panel Studies

**DOI:** 10.1093/joccuh/uiag046

**Published:** 2026-08-13

**Authors:** Wenshuai Qin, Hao Huang, Rui Li, Ziyodulla Nurov

**Affiliations:** Faculty of Economics and Tourism, Bukhara State University, 11M Iqbal Street, Bukhara City, Bukhara State 200117, Uzbekistan; School of Economics and Management, Anhui Vocational College of Electronics & Information Technology, No. 1000 Caoshan Road, Longzihu District, Bengbu City, Anhui Province 233300, China; Graduate School of Business Management, Philippine Christian University, 1648 Taft Ave, Malate, Manila 1004, Philippines; Faculty of Economics and Tourism, Bukhara State University, 11M Iqbal Street, Bukhara City, Bukhara State 200117, Uzbekistan

**Keywords:** employment quality, workers’ health, well-being, occupational health, fixed-effects model, China Family Panel Studies

## Abstract

**Objectives:**

This study examined the relationship between employment quality and workers’ health and well-being in China.

**Methods:**

Data were drawn from the 2018, 2020, and 2022 waves of the China Family Panel Studies. We constructed an individual-level panel sample and measured employment quality using 4 dimensions: reward, job satisfaction, working-time quality, and social security. Individual fixed-effects models were used as the main estimation approach. Robustness checks were conducted using a balanced panel sample and an alternative employment quality index excluding the job satisfaction dimension.

**Results:**

Higher employment quality was positively associated with better self-rated health, mental health, happiness, and confidence in the future. Among the 4 dimensions, job satisfaction showed the most consistent positive associations with all 4 outcomes, whereas reward and working-time quality were mainly associated with mental health. Social security did not show statistically significant associations in the fixed-effects models. In the robustness check excluding job satisfaction, the coefficients of the alternative employment quality index remained positive but became smaller and less statistically significant; only the association with mental health remained statistically significant.

**Conclusions:**

Workers’ health and well-being were related not only to employment status or income, but also to broader aspects of employment quality, including subjective work experience, working-time arrangements, and institutional protection. Employment quality and its different dimensions should receive greater attention in occupational health promotion and the evaluation of high-quality employment.

## Teaser

**Key points**
 

- **What is already known on this topic:** Employment status and income alone do not fully explain differences in workers’ health and well-being. Poor job quality and precarious employment have been linked to adverse health outcomes, but longitudinal evidence on multiple dimensions of employment quality and multiple health and well-being outcomes among Chinese workers remains limited.
- **What this study adds:** Using 3 waves of nationally representative China Family Panel Studies data, this study showed that employment quality was positively associated with self-rated health, mental health, happiness, and confidence in the future. Job satisfaction was the most consistent dimension across all outcomes, while reward and working-time quality were mainly associated with mental health. In the robustness check excluding job satisfaction, only the association with mental health remained statistically significant.
- **How this study might affect research, practice, or policy:** Occupational health promotion should move beyond a narrow focus on employment status, income, or traditional workplace hazards. Improving work experience, managing working-time arrangements, and strengthening institutional protection may be important pathways for promoting workers’ health and well-being.

## 1. Introduction

In occupational health and public health research, employment status alone cannot fully explain differences in workers’ health; employment quality also matters. Studies on precarious employment and job quality link employment insecurity, insufficient income, work stress, and lack of labor rights and social protection to poorer health and well-being outcomes.^1–5^ Workplace mental health research further indicates that excessive workload, low job control, job insecurity, and poor organizational environments increase the risk of mental health problems.^6^^,^^7^ Job quality also explains variations in overall well-being to a degree comparable to health and more strongly than several common individual characteristics.^8^ Together, these findings show that employment status and income are insufficient explanations.

In China, the policy goal of high-quality and full employment has shifted attention from employment quantity to employment quality. The 2024 Opinions of the CPC Central Committee and the State Council on Implementing the Employment-First Strategy to Promote High-Quality and Full Employment emphasize “stable jobs, reasonable income, reliable security, and occupational safety,” as well as workers’ sense of gain, happiness, and security.^9^ Recent evidence also points to employment-quality challenges in China, including long or mismatched working hours, income disparities, unequal access to social security, and variation in job satisfaction.^10–13^ These policy priorities and labor market conditions make China an important setting for examining employment quality and workers’ health and well-being.

Researchers have refined the concept and measurement of employment quality over time. Early studies focused mainly on objective job characteristics, such as earnings, employment security, and working conditions, while later frameworks conceptualized employment quality as multidimensional.^14–16^ For example, the OECD framework emphasizes earnings quality, labor market security, and the quality of the working environment, whereas the Eurofound framework includes broader dimensions such as working-time quality, work intensity, skills and autonomy, career prospects, and the social and physical work environment.^14–16^

As research has progressed, scholars have increasingly recognized that objective job characteristics alone cannot fully capture workers’ employment experiences. Recent studies therefore assess employment quality using both objective and subjective indicators.^17–19^ Objective measures include earnings, working time, employment security, and social protection, whereas subjective measures capture workers’ evaluations of their employment conditions. Objective and subjective indicators capture complementary aspects of employment quality and provide a more comprehensive assessment.^17–19^

Building on these conceptual and measurement developments, studies have examined how employment quality relates to workers’ health and well-being. Existing evidence suggests that favorable employment conditions correspond to better health and well-being, whereas poor job quality and precarious employment are linked to poorer self-rated health, worse mental health, emotional distress, and lower overall well-being.^1–5^^,^^18–20^ Recent studies have extended this research to happiness, job-related well-being, and quality of life.^8^^,^^19^^,^^21^ Different dimensions may matter through different pathways: reward may improve material resources, working-time quality may support recovery, social security may reduce uncertainty, and job satisfaction may capture workers’ evaluations of work.^13–19^

Despite this growing evidence, several questions remain. First, many studies examine employment quality overall, while less is known about dimension-specific relationships with health and well-being outcomes.^18–20^ Second, previous studies often focused on a single health or well-being outcome, leaving limited evidence on multiple worker outcomes within the same analytical framework.^13^^,^^21–24^ Third, longitudinal evidence linking multiple dimensions of employment quality to multiple health and well-being outcomes among Chinese workers remains limited.^13^^,^^22–24^ Examining both the overall employment quality index and its individual dimensions can therefore clarify how different aspects of employment quality relate to workers’ health and well-being.

Against this background, this study used data from the 2018, 2020, and 2022 waves of the China Family Panel Studies (CFPS) to examine the relationship between employment quality and workers’ health and well-being. Based on previous literature and CFPS data availability, employment quality was measured using 4 dimensions: reward, job satisfaction, working-time quality, and social security. The analysis considered 4 outcomes: self-rated health, mental health, happiness, and confidence in the future. It examined both the overall employment quality index and its individual dimensions using individual fixed-effects models, with robustness checks based on a balanced panel sample and an alternative index excluding job satisfaction. By integrating multiple employment quality dimensions and multiple health and well-being outcomes, this study provides new longitudinal evidence from China.

## 2. Methods

### 2.1 Data source and sample construction

This study used data from the 2018, 2020, and 2022 waves of the CFPS, a nationally representative longitudinal survey conducted by the Institute of Social Science Survey at Peking University. We linked respondents across waves using unique identification codes and constructed an individual-level unbalanced panel.

The pooled dataset contained 88 200 person-year observations from the 3 waves and included all respondents interviewed in each wave, not only employed respondents. To construct the employment quality index, we retained observations with valid information on all 4 dimensions: reward, job satisfaction, working-time quality, and social security, reducing the sample to 26 312 person-year observations. After excluding observations with missing control variables, the common analytical sample for descriptive analyses included 25 271 person-year observations. Table S1 presents the complete sample selection process.

We estimated separate fixed-effects models for each outcome because each model required valid information on the corresponding outcome variable. The final estimation samples therefore differed slightly across models, ranging from 25 128 person-year observations for mental health to 25 182 for happiness.

We used publicly available anonymized secondary data from the CFPS. All participants provided informed consent before the original survey, and this secondary analysis did not involve direct interaction with participants or require additional ethical approval.

### 2.2 Measures

The main variables in this study included the explanatory variable, outcome variables, and control variables. The explanatory variable was the employment quality index. The outcome variables captured workers’ health and well-being. The control variables included individual characteristics that may relate to employment quality, health, and well-being. Table 1 presents the definitions and measurements of all variables.

**Table 1:** Variable definitions and measurements.

Variable	Survey item	Coding/measurement
**Employment quality index**	Constructed from the reward, job satisfaction, working-time quality, and social security indicators	The 4 indicators were standardized and averaged. A higher value indicates higher employment quality
**Reward indicator**	Including all wages, bonuses, cash benefits, and in-kind subsidies, and deducting taxes and social insurance and housing provident fund contributions, how much did you receive from this job in total over the past 12 months?	Measured by annual labor income from the job. The value is log-transformed and then standardized. A higher value indicates a higher level of reward
**Job satisfaction indicator**	How satisfied are you with the income from this job? How satisfied are you with the security of this job? How satisfied are you with the working environment of this job? How satisfied are you with the working time of this job? How satisfied are you with the promotion opportunities of this job? Overall, how satisfied are you with this job?	Each item is coded as: 1 = very dissatisfied; 2 = somewhat dissatisfied; 3 = neutral; 4 = somewhat satisfied; 5 = very satisfied. After all items were coded in the same direction, the mean value was calculated and standardized. A higher value indicates higher job satisfaction
**Working-time quality indicator**	How many hours do you usually work per week in your current job?	Weekly working hours are grouped and coded as: 1 = >55 hours; 2 = 49-55 hours; 3 = 41-48 hours; 4 = ≤40 hours. The indicator is then standardized. A higher value indicates higher working-time quality
**Social security indicator**	What types of insurance are provided by this job? Does this job provide a housing provident fund?	Pension insurance, medical insurance, unemployment insurance, work-related injury insurance, maternity insurance, and housing provident fund are each coded as 1 if provided and 0 otherwise. The mean value is calculated and then standardized. A higher value indicates a higher level of social security
**Alternative employment quality index excluding job satisfaction**	Constructed from 3 dimensions of employment quality: reward, working-time quality, and social security	This index was used for robustness checks. The 3 dimensions were standardized and then averaged, excluding job satisfaction. A higher value indicates higher employment quality excluding the job satisfaction dimension
**Self-rated health**	How would you rate your health status?	The original item is coded as: 1 = very healthy; 2 = healthy; 3 = relatively healthy; 4 = fair; 5 = unhealthy. It was reverse-coded in the empirical analysis as 1 = poorest and 5 = best. A higher value indicates better self-rated health
**Mental health**	Constructed from 8 CES-D items: I felt depressed; I felt that everything I did was an effort; my sleep was poor; I felt happy; I felt lonely; I enjoyed life; I felt sad; and I felt that I could not go on with my life	Each item is coded as: 1 = rarely or none of the time, less than 1 day; 2 = some of the time, 1-2 days; 3 = often, 3-4 days; 4 = most of the time, 5-7 days. After item directions are unified, the mean value is calculated. A higher value indicates better mental health
**Happiness**	Respondent’s reported level of overall happiness	Measured on a 0-10 scale. A higher value indicates a higher level of happiness
**Confidence in the future**	Confidence in one’s own future	Measured on a 1-5 scale. A higher value indicates greater confidence in the future
**Gender**	Respondent’s gender	0 = female; 1 = male
**Age**	Age	Continuous variable, measured in years
**Educational attainment**	Highest level of education completed	1 = illiterate or semi-literate; 2 = primary school; 3 = junior high school; 4 = senior high school, technical secondary school, technical school, or vocational high school; 5 = junior college; 6 = bachelor’s degree; 7 = master’s degree; 8 = doctoral degree
**Marital status**	Constructed based on marital status at the most recent interview	1 = married or cohabiting; 0 = unmarried, divorced, or widowed
**Rural household registration status**	Respondent’s household registration status under China’s hukou system^a^	1 = agricultural hukou; 0 = nonagricultural hukou or resident hukou. This variable identifies whether the respondent held a rural household registration

Abbreviation: CES-D, Center for Epidemiologic Studies Depression Scale. ^a^Hukou refers to China’s household registration system.

#### 2.2.1 Employment quality

We used the employment quality index as the explanatory variable. Based on previous research and the availability of CFPS data, we measured employment quality using 4 dimensions: reward, job satisfaction, working-time quality, and social security. These dimensions captured workers’ economic rewards, subjective work experience, working-time arrangements, and institutional protection.

Reward was measured using respondents’ annual after-tax labor income from the current job, which was log-transformed and standardized. Job satisfaction was calculated from 6 items covering satisfaction with income, job security, work environment, working time, promotion opportunities, and the job as a whole. Working-time quality was constructed from weekly working hours, coded as 1 for more than 55 hours, 2 for 49-55 hours, 3 for 41-48 hours, and 4 for 40 hours or less, and then standardized. Social security was measured using 6 employer-provided benefits: pension insurance, medical insurance, unemployment insurance, work-related injury insurance, maternity insurance, and the housing provident fund. Each benefit was coded as 1 if provided and 0 otherwise; the mean value was then calculated and standardized.

After aligning the direction of the 4 dimensions and standardizing them, we calculated their arithmetic mean to form the employment quality index. Higher values indicate better employment quality. For the robustness analysis, we also constructed an alternative employment quality index excluding job satisfaction by averaging reward, working-time quality, and social security.

#### 2.2.2 Outcome variables

The outcome variables covered workers’ health and well-being. Health outcomes included self-rated health and mental health. Self-rated health came from respondents’ overall assessment of their health status. We reverse-coded this variable so that higher values indicated better self-rated health. We measured mental health using 8 items from the Center for Epidemiologic Studies Depression Scale (CES-D) in the CFPS. After aligning the item directions, we calculated the mean score, with higher values indicating better mental health.

Well-being outcomes included happiness and confidence in the future. Happiness was measured on a 0-10 scale, with higher values indicating higher levels of happiness. Confidence in the future was measured on a 1-5 scale, with higher values indicating greater confidence in the future. Table 1 provides detailed measurement information. Because the 4 outcome variables used different scale ranges, their descriptive statistics should be interpreted within their respective scales.

#### 2.2.3 Control variables

The control variables included age, educational attainment, marital status, and rural household registration status. These variables may relate to workers’ employment quality, health, and well-being, so we included them in the models. Rural household registration status refers to respondents’ household registration status under China’s hukou system. We coded agricultural hukou as 1 and nonagricultural hukou or resident hukou as 0. Table 1 presents the detailed coding information. We used gender in the sample description but did not include it in the individual fixed-effects models because gender remains largely time-invariant during the observation period and is absorbed by individual fixed effects.

### 2.3 Statistical analysis

To examine the relationship between employment quality and workers’ health and well-being, we constructed an individual-level panel sample using data from the 2018, 2020, and 2022 waves of the CFPS and specified the following baseline model:


(1) \begin{eqnarray*} {Y}_{it}=\mathrm{\beta} E{Q}_{it}+\mathrm{\gamma} {X}_{it}+{\mathrm{\mu}}_i+{\mathrm{\lambda}}_t+{\mathrm{\varepsilon}}_{it} \end{eqnarray*}

where ${Y}_{it}$denotes the outcome variable for individual *i* in year *t*, including self-rated health, mental health, happiness, and confidence in the future; $E{Q}_{it}$denotes the employment quality index; ${X}_{it}$represents the control variables, including age, educational attainment, marital status, and rural household registration status; ${\mu}_i$denotes individual fixed effects; ${\lambda}_t$denotes year fixed effects; and ${\varepsilon}_{it}$ is the error term. The coefficient *β* is the key parameter of interest and captures the association between changes in employment quality and changes in workers’ health and well-being outcomes.

Because the same respondents were observed repeatedly across different years, we used individual fixed-effects models as the baseline estimation method. This approach controls for time-invariant individual characteristics and focuses on the relationship between within-individual changes in employment quality and changes in the outcome variables. We used robust SEs clustered at the individual level in all models. Time-invariant variables, such as gender, were absorbed by the individual fixed effects and therefore did not enter the coefficient estimation.

To compare the roles of different dimensions of employment quality, we further estimated the following subdimension model:


(2) \begin{eqnarray*} {Y}_{it}={\beta}_1{R}_{it}+{\beta}_2J{S}_{it}+{\beta}_3 WT{Q}_{it}+{\beta}_4S{S}_{it}+\gamma{X}_{it}+{\mu}_i+{\lambda}_t+{\varepsilon}_{it} \end{eqnarray*}

where ${R}_{it}$, $J{S}_{it}$, $WT{Q}_{it}$, and $S{S}_{it}$ denote the reward, job satisfaction, working-time quality, and social security indicators, respectively. The other terms have the same meanings as in Equation 1. This model examines whether different dimensions of employment quality show different associations with workers’ health and well-being outcomes.

We conducted 2 robustness checks. First, we restricted the sample to respondents who were observed in all 3 survey waves in 2018, 2020, and 2022 to assess whether the unbalanced panel structure affected the main results. Second, we excluded the job satisfaction dimension and reconstructed an alternative employment quality index based on reward, working-time quality, and social security. This test assessed the sensitivity of the findings to the inclusion of the subjective job satisfaction dimension. These robustness checks allowed us to assess the stability of the association between employment quality and workers’ health and well-being outcomes.

## 3. Results

### 3.1 Sample selection

This study constructed a panel dataset using the 2018, 2020, and 2022 waves of the CFPS. The original pooled sample included 88 200 person-year observations: 32 669 from 2018, 28 530 from 2020, and 27 001 from 2022. The large reduction in sample size occurred because the original pooled sample included all respondents interviewed in each wave, whereas the analytical sample required valid information on employment quality, control variables, and the corresponding health and well-being outcomes.

Table S1 presents the sample selection procedure. To construct the employment quality index, we first retained observations with valid information on all 4 employment quality dimensions: reward, job satisfaction, working-time quality, and social security. This step yielded 26 312 person-year observations. After excluding observations with missing control variables, the descriptive analytical sample included 25 271 person-year observations. The final estimation samples differed slightly across outcome models because each model also required valid information on its corresponding outcome variable: 25 140 observations for self-rated health, 25 128 for mental health, 25 182 for happiness, and 25 158 for confidence in the future.

### 3.2 Descriptive statistics

Table 2 reports the demographic and socioeconomic characteristics of the descriptive analytical sample. The sample included 25 271 person-year observations with valid information on all 4 employment quality dimensions and the control variables. The distribution across survey waves was relatively balanced, with 37.07% of observations from 2018, 31.23% from 2020, and 31.70% from 2022. Most respondents were between 25 and 54 years old, with the largest age group being 25-34 years (33.26%). Men accounted for 57.56% of the sample, married or cohabiting respondents accounted for 75.06%, and respondents with rural household registration accounted for 63.33%. Because these characteristics are categorical, Table 2 presents them using frequencies and percentages.

**Table 2:** Sample characteristics.

Variable	Category	Frequency	Percentage
**Year**	2018	9367	37.07
	2020	7893	31.23
	2022	8011	31.70
**Age group**	16-24 years	2474	9.79
	25–34 years	8404	33.26
	35-44 years	5913	23.40
	45-54 years	5471	21.65
	55-64 years	2425	9.60
	65 years and above	584	2.31
**Gender**	Female	10726	42.44
	Male	14545	57.56
**Educational attainment**	Illiterate or semi-literate	1298	5.14
	Primary school	2982	11.80
	Junior high school	7674	30.37
	Senior high school, technical secondary school, technical school, or vocational high school	5024	19.88
	Junior college	4060	16.07
	Bachelor’s degree	3860	15.27
	Master’s degree	343	1.36
	Doctoral degree	30	0.12
**Marital status**	Unmarried, divorced, or widowed	6303	24.94
	Married or cohabiting	18968	75.06
**Rural household registration status**	Nonrural household registration	9267	36.67
	Rural household registration	16004	63.33

This table is based on the descriptive baseline sample with valid information on the 4 employment quality dimensions and complete control variables, including 25 271 observations. Different regression models further required valid information on the corresponding outcome variable; therefore, the final sample size varies slightly across models.

Table 3 presents the descriptive statistics for the main variables. The employment quality dimensions and the employment quality index are standardized relative measures rather than original measurement units. The employment quality index had a mean of −0.012 and an SD of 0.577. The means of reward, job satisfaction, working-time quality, and social security were 0.049, −0.030, −0.131, and 0.063, respectively. These values do not necessarily equal zero because Table 3 is based on the common analytical sample, whereas standardization used the valid sample for each original dimension. The employment quality index had a smaller SD than the individual dimensions because it averaged the 4 standardized dimensions.

**Table 3:** Descriptive statistics of main variables.

Variable	*n*	Mean	SD	Min	25th percentile	Median	75th percentile	Max
**Reward**	25 271	0.049	0.934	−6.438	−0.123	0.227	0.483	2.366
**Job satisfaction**	25 271	−0.030	0.926	−3.495	−0.618	0.141	0.521	1.805
**Working-time quality**	25 271	−0.131	0.964	−1.144	−1.126	−0.396	1.065	1.107
**Social security**	25 271	0.063	0.995	−1.112	−0.982	0.259	1.024	1.370
**Employment quality index**	25 271	−0.012	0.577	−2.912	−0.421	−0.002	0.442	1.432
**Self-rated health**	25 140	3.272	1.043	1.000	3.000	3.000	4.000	5.000
**Mental health**	25 128	3.335	0.465	1.000	3.000	3.375	3.750	4.000
**Happiness**	25 182	7.434	1.980	0.000	6.000	8.000	9.000	10.000
**Confidence in the future**	25 158	4.108	0.879	1.000	4.000	4.000	5.000	5.000

Descriptive statistics are based on the baseline sample with valid information on the 4 employment quality dimensions and complete control variables. The sample size varies slightly across outcome variables because of missing values in the corresponding outcomes. Reward, job satisfaction, working-time quality, social security, and the employment quality index were standardized or constructed from standardized indicators. Higher values indicate higher employment quality, better health, or higher well-being.

The mean values of self-rated health, mental health, happiness, and confidence in the future were 3.272, 3.335, 7.434, and 4.108, respectively. These values should be interpreted within their own scales: 1-5 for self-rated health and confidence in the future, 1-4 for mental health, and 0-10 for happiness. Therefore, the means should not be directly compared across outcomes. Higher values indicate better health or well-being. The number of valid observations differed slightly across outcomes because of item-specific missing values, so the fixed-effects models used the valid sample for each corresponding outcome.

### 3.3 Baseline fixed-effects regression results

Table 4 reports the baseline fixed-effects regression results for the association between the employment quality index and workers’ health and well-being. The 4 outcome variables were self-rated health, mental health, happiness, and confidence in the future. All models controlled for age, educational attainment, marital status, rural household registration status, year fixed effects, and individual fixed effects. Robust SEs were clustered at the individual level.

**Table 4:** Baseline fixed-effects regression results.

Variable	Self-rated health	Mental health	Happiness	Confidence in the future
**Employment quality index**	0.1238^***^ (0.0230)	0.0660^***^ (0.0098)	0.1802^***^ (0.0396)	0.0933^***^ (0.0191)
**Age**	−0.0400 (0.0789)	−0.0030 (0.0243)	−0.2640^**^ (0.1193)	−0.1139^**^ (0.0464)
**Educational attainment**	−0.0056 (0.0329)	0.0253^**^ (0.0127)	0.0103 (0.0568)	0.0014 (0.0267)
**Marital status**	−0.0203 (0.0542)	−0.0238 (0.0246)	−0.0832 (0.1163)	−0.0276 (0.0461)
**Rural household registration status**	0.0027 (0.0401)	−0.0078 (0.0173)	−0.0484 (0.0734)	−0.0265 (0.0351)
**2020**	0.1044 (0.1587)	−0.0032 (0.0493)	0.4394^*^ (0.2399)	0.1797^*^ (0.0938)
**2022**	0.1566 (0.3161)	−0.0262 (0.0976)	0.8795^*^ (0.4779)	0.3699^**^ (0.1862)
**Constant**	4.7889 (2.9304)	3.3883^***^ (0.9026)	17.3777^***^ (4.4339)	8.4123^***^ (1.7253)
**Observations**	25 140	25 128	25 182	25 158
**Number of individuals**	14 688	14 686	14 709	14 699
**Within *R*** ^**2**^	0.0039	0.0087	0.0066	0.0070

This table reports estimates from individual fixed-effects models. Robust SEs clustered at the individual level are shown in parentheses. ^***^P < .01, ^**^P < .05, ^*^P < .10. Each model was estimated using the valid analytical sample for the corresponding outcome variable. Gender was not included in the fixed-effects models because it is largely time-invariant at the individual level.

The employment quality index showed positive and statistically significant associations with all 4 outcomes. The coefficients were 0.1238 for self-rated health, 0.0660 for mental health, 0.1802 for happiness, and 0.0933 for confidence in the future, all statistically significant at the 1% level. These results indicate that, after controlling for individual fixed effects and other covariates, higher employment quality corresponded to better self-rated health, better mental health, higher happiness, and greater confidence in the future.

Overall, the baseline fixed-effects results suggest that within-individual improvements in employment quality were consistently associated with improvements in workers’ health and well-being outcomes.

### 3.4 Fixed-effects regression results for employment quality subdimensions

After examining the overall employment quality index, we decomposed employment quality into 4 dimensions: reward, job satisfaction, working-time quality, and social security. Table 5 reports the corresponding fixed-effects results.

**Table 5:** Fixed-effects regression results for the subdimensions of employment quality.

Variable	Self-rated health	Mental health	Happiness	Confidence in the future
**Reward indicator**	−0.0095 (0.0111)	0.0097^**^ (0.0046)	−0.0062 (0.0208)	0.0097 (0.0092)
**Job satisfaction indicator**	0.1190^***^ (0.0125)	0.0386^***^ (0.0055)	0.1712^***^ (0.0234)	0.0941^***^ (0.0108)
**Working-time quality indicator**	0.0109 (0.0113)	0.0138^***^ (0.0050)	−0.0106 (0.0212)	−0.0105 (0.0095)
**Social security indicator**	0.0032 (0.0117)	0.0032 (0.0052)	0.0261 (0.0221)	−0.0022 (0.0105)
**Age**	−0.0219 (0.0717)	0.0007 (0.0238)	−0.2391^**^ (0.1208)	−0.1035^**^ (0.0459)
**Educational attainment**	−0.0001 (0.0329)	0.0270^**^ (0.0128)	0.0168 (0.0568)	0.0052 (0.0266)
**Marital status**	−0.0173 (0.0543)	−0.0234 (0.0247)	−0.0781 (0.1157)	−0.0253 (0.0463)
**Rural household registration status**	0.0026 (0.0396)	−0.0083 (0.0172)	−0.0464 (0.0735)	−0.0257 (0.0349)
**2020**	0.0700 (0.1443)	−0.0106 (0.0482)	0.3939 (0.2429)	0.1607^*^ (0.0929)
**2022**	0.0838 (0.2872)	−0.0418 (0.0956)	0.7813 (0.4839)	0.3277^*^ (0.1844)
**Constant**	4.0950 (2.6629)	3.2447^***^ (0.8836)	16.4232^***^ (4.4899)	8.0103^***^ (1.7095)
**Observations**	25 140	25 128	25 182	25 158
**Number of individuals**	14 688	14 686	14 709	14 699
**Within R** ^**2**^	0.0119	0.0114	0.0114	0.0136

This table reports estimates from individual fixed-effects models. Robust SEs clustered at the individual level are shown in parentheses. ^***^P < .01, ^**^P < .05, ^*^P < .10. Reward, job satisfaction, working-time quality, and social security are standardized employment quality subdimension variables. Gender was not included in the fixed-effects models because it is largely time-invariant at the individual level.

Job satisfaction showed the most consistent associations. It was positively associated with all 4 outcomes at the 1% level. A 1-unit increase in job satisfaction corresponded to increases of 0.1190 in self-rated health, 0.0386 in mental health, 0.1712 in happiness, and 0.0941 in confidence in the future. These results suggest that within-individual improvements in job satisfaction were consistently linked to better health and well-being outcomes.

Reward and working-time quality showed more limited associations. Both were statistically significant only in the mental health model, with coefficients of 0.0097 and 0.0138, respectively. Neither dimension showed statistically significant associations with self-rated health, happiness, or confidence in the future.

Social security did not reach statistical significance in any model. This result should not be interpreted as evidence that social security is unimportant. In fixed-effects models, estimates depend on within-individual changes over time, and social security coverage may have changed little during the observation period. The indicator also measured only whether employers provided benefits, without capturing benefit levels, contribution quality, or actual use.

Overall, the subdimension analysis showed that employment quality dimensions differed in their associations with health and well-being outcomes. Job satisfaction showed the broadest and most consistent associations, reward and working-time quality were mainly associated with mental health, and social security did not show stable statistically significant associations.

### 3.5 Robustness checks

To assess the stability of the baseline results, we conducted 2 robustness checks. First, we restricted the sample to respondents observed in all 3 survey waves in 2018, 2020, and 2022 to examine whether the unbalanced panel structure affected the main findings. Second, we reconstructed an alternative employment quality index excluding job satisfaction by averaging the 3 standardized dimensions of reward, working-time quality, and social security.

Table 6 reports the robustness check results. In Panel A, based on the balanced panel sample, the employment quality index remained positively and statistically significantly associated with all 4 outcomes. The coefficients were 0.1311 for self-rated health, 0.0647 for mental health, 0.1308 for happiness, and 0.0854 for confidence in the future, all significant at the 1% level. These results were consistent with the baseline findings and suggest that the main results were not driven by the unbalanced panel structure.

**Table 6:** Robustness checks.

Variable	Self-rated health	Mental health	Happiness	Confidence in the future
**Panel A. Balanced panel sample**				
** Employment quality index**	0.1311^***^ (0.0261)	0.0647^***^ (0.0113)	0.1308^***^ (0.0443)	0.0854^***^ (0.0219)
** Age**	0.1386 (0.0985)	−0.0006 (0.0513)	−0.3047 (0.2365)	−0.0936 (0.1104)
** Educational attainment**	0.0209 (0.0392)	0.0314^**^ (0.0141)	−0.0277 (0.0637)	0.0259 (0.0303)
** Marital status**	0.0233 (0.0637)	−0.0500^*^ (0.0289)	0.0125 (0.1423)	−0.0553 (0.0529)
** Rural household registration status**	0.0080 (0.0473)	−0.0104 (0.0202)	−0.0492 (0.0816)	−0.0398 (0.0395)
** 2020**	−0.2534 (0.1980)	−0.0116 (0.1032)	0.5368 (0.4741)	0.1373 (0.2215)
** 2022**	−0.5487 (0.3943)	−0.0354 (0.2055)	1.0733 (0.9467)	0.2917 (0.4422)
** Constant**	−2.0488 (3.6905)	3.3054^*^ (1.9211)	19.0786^**^ (8.8619)	7.6165^*^ (4.1373)
** Observations**	14 699	14 693	14 709	14 699
** Number of individuals**	6722	6721	6724	6722
** Within *R*** ^**2**^	0.0044	0.0086	0.0043	0.0062
**Panel B. Employment quality index excluding job satisfaction**				
** Employment quality index excluding job satisfaction**	0.0190 (0.0198)	0.0322^***^ (0.0083)	0.0317 (0.0352)	0.0124 (0.0164)
** Age**	−0.0352 (0.0769)	−0.0026 (0.0242)	−0.2573^**^ (0.1205)	−0.1100^**^ (0.0473)
** Educational attainment**	−0.0020 (0.0331)	0.0261^**^ (0.0127)	0.0153 (0.0567)	0.0044 (0.0267)
** Marital status**	−0.0253 (0.0543)	−0.0257 (0.0246)	−0.0904 (0.1165)	−0.0315 (0.0462)
** Rural household registration status**	0.0034 (0.0401)	−0.0076 (0.0173)	−0.0472 (0.0734)	−0.0258 (0.0351)
** 2020**	0.0953 (0.1548)	−0.0040 (0.0491)	0.4271^*^ (0.2424)	0.1725^*^ (0.0957)
** 2022**	0.1389 (0.3081)	−0.0273 (0.0973)	0.8553^*^ (0.4828)	0.3558^*^ (0.1900)
** Constant**	4.5962 (2.8567)	3.3703^***^ (0.8996)	17.1130^***^ (4.4793)	8.2591^***^ (1.7610)
** Observations**	25 140	25 128	25 182	25 158
** Number of individuals**	14 688	14 686	14 709	14 699
** Within *R*** ^**2**^	0.0006	0.0053	0.0046	0.0046

This table reports robustness checks using individual fixed-effects models. Panel A reports estimates based on the balanced panel sample, defined as respondents observed in all 3 survey waves in 2018, 2020, and 2022. Panel B reports estimates using an alternative employment quality index that excludes the job satisfaction dimension. This alternative index was constructed by averaging the standardized reward, working-time quality, and social security indicators. Robust SEs clustered at the individual level are shown in parentheses. ^***^P < .01, ^**^P < .05, ^*^P < .10. Gender was not included in the fixed-effects models because it is largely time-invariant at the individual level.

In Panel B, using the alternative employment quality index excluding job satisfaction, the coefficients remained positive for all 4 outcomes, but the associations became weaker and less statistically significant than in the baseline models. Only the association with mental health remained statistically significant, with a coefficient of 0.0322 at the 1% level. The associations with self-rated health, happiness, and confidence in the future did not reach statistical significance. These results suggest that job satisfaction is an important component of employment quality and that the relationship between employment quality and health and well-being varies across dimensions and outcomes.

## 4. Discussion

Using longitudinal data from the 2018, 2020, and 2022 waves of the CFPS, this study examined the relationship between employment quality and workers’ health and well-being in China. Higher employment quality was positively associated with self-rated health, mental health, happiness, and confidence in the future. The subdimension analysis showed that job satisfaction had the most consistent associations with all 4 outcomes, whereas reward and working-time quality were mainly associated with mental health. Social security did not show statistically significant associations in the fixed-effects models. The robustness checks supported the baseline findings in the balanced panel sample but showed weaker and more outcome-specific associations after job satisfaction was excluded.

These findings are consistent with previous research linking poor job quality and precarious employment to worse health and well-being outcomes, and with studies emphasizing the multidimensional nature of employment quality. This study extends the literature by providing longitudinal evidence from Chinese workers and by examining multiple outcomes within the same framework. The results suggest that workers’ health and well-being cannot be understood only through employment status or income; work experience, working-time arrangements, and institutional protection also matter.

Job satisfaction emerged as the most stable employment quality dimension. Because this indicator captured workers’ evaluations of income, job security, work environment, working time, promotion opportunities, and the job as a whole, it may reflect workers’ overall work experience. However, this finding requires careful interpretation because job satisfaction is subjective and may be closer to well-being outcomes than more objective dimensions. The robustness check excluding job satisfaction supports this interpretation: the alternative index remained positively associated with all outcomes, but only mental health remained statistically significant.

Reward and working-time quality showed statistically significant associations only with mental health, suggesting that mental health may respond more sensitively to changes in economic pressure, work intensity, rest, and recovery conditions. By contrast, self-rated health may reflect longer-term health accumulation, while happiness and confidence in the future may depend more on family resources, social relationships, assets, and broader expectations. The nonsignificant results for social security should not be interpreted as evidence that institutional protection is unimportant. In fixed-effects models, estimates depend on within-individual changes over time, and changes in social security coverage may have been limited. The indicator also measured only whether employers provided benefits, not contribution levels, benefit adequacy, accessibility, or actual use.

### 4.1 Limitations

This study has several limitations. First, the measurement of employment quality was constrained by the employment-related items available in the CFPS. The index captured reward, job satisfaction, working-time quality, and social security, but did not include dimensions such as labor contracts, job autonomy, occupational risk exposure, organizational support, or career development opportunities. Second, job satisfaction is a subjective dimension and may be conceptually closer to well-being outcomes than the other dimensions. Although the robustness check excluding job satisfaction partly addressed this concern, future research could further distinguish subjective and objective dimensions of employment quality. Third, the health and well-being outcomes relied mainly on self-reported data, so reporting bias and differences in subjective perception could not be fully excluded. Fourth, although individual fixed-effects models controlled for time-invariant individual characteristics, time-varying omitted variables and reverse causality may remain. The findings should therefore be interpreted as associations rather than strong causal evidence. Finally, this study used only 3 survey waves and did not examine group heterogeneity or long-term lagged relationships.

## 5. Conclusions

From an occupational health perspective, these findings suggest that health promotion should not focus only on employment status, income, or traditional workplace hazards. Improving workers’ health and well-being may require attention to work experience, excessive working hours, fair rewards, and the quality and accessibility of social security. For policymakers and employers, the results highlight the importance of high-quality employment and management practices that improve job satisfaction, protect recovery time, and provide stable institutional support.

## Supplementary Material

Supplementary_materials_uiag046

## Data Availability

The data used in this study are from the China Family Panel Studies (CFPS), conducted by the Institute of Social Science Survey at Peking University. The CFPS data are publicly available upon application through the official CFPS data platform. The statistical code used in this study is available from the corresponding author upon reasonable request.
