# Supplementary material for: Employment quality and workers’ health and well-being in China: longitudinal evidence from the China Family Panel Studies
Source: J Occup Health. 2026 Aug 13;68(1):uiag046. doi: 10.1093/joccuh/uiag046 (PMC13540727; doi:10.1093/joccuh/uiag046)
Supplement: Supplementary file 1 [file supplementary_materials_uiag046.zip › STROBE Checklist.docx]

**STROBE Statement Checklist for Cohort Studies**

This checklist was completed based on the STROBE recommendations for reporting observational studies. The present study used longitudinal panel data from the China Family Panel Studies and is reported as an observational cohort-style panel study.

| **No.** | **STROBE Recommendation** | **Location in Manuscript** |
| --- | --- | --- |
| 1 | Indicate the study design with a commonly used term in the title or abstract, and provide an informative summary of what was done and found. | Title; Abstract |
| 2 | Explain the scientific background and rationale for the investigation. | Introduction, paragraphs 1–5 |
| 3 | State specific objectives, including any prespecified hypotheses. | Introduction, final paragraph |
| 4 | Present key elements of study design early in the paper. | Methods, Section 2.1; Section 2.3 |
| 5 | Describe the setting, locations, and relevant dates, including periods of recruitment, exposure, follow-up, and data collection. | Methods, Section 2.1 |
| 6 | Give the eligibility criteria, sources, and methods of selection of participants. | Methods, Section 2.1 |
| 7 | Define all outcomes, exposures, predictors, potential confounders, and effect modifiers. | Methods, Section 2.2; Table 1 |
| 8 | Give sources of data and details of methods of assessment for each variable. | Methods, Section 2.2; Table 1 |
| 9 | Describe any efforts to address potential sources of bias. | Methods, Section 2.3; Results, Section 3.5; Limitations |
| 10 | Explain how the study size was arrived at. | Methods, Section 2.1; Supplementary Table S1 |
| 11 | Explain how quantitative variables were handled in the analyses. | Methods, Section 2.2; Table 1 |
| 12 | Describe all statistical methods, including methods used to control for confounding, subgroup or sensitivity analyses, and handling of missing data. | Methods, Section 2.3; Results, Sections 3.2 and 3.5 |
| 13 | Report numbers of individuals at each stage of the study. | Methods, Section 2.1; Results, Section 3.1; Supplementary Table S1 |
| 14 | Give characteristics of study participants. | Results, Section 3.2; Table 2 |
| 15 | Report numbers of outcome events or summary measures. | Results, Section 3.2; Table 3 |
| 16 | Give unadjusted and adjusted estimates and their precision, and make clear which confounders were adjusted for. | Results, Sections 3.3–3.5; Tables 4–6 |
| 17 | Report other analyses, such as subgroup, interaction, and sensitivity analyses. | Results, Section 3.5; Table 6. Robustness checks were reported; no subgroup or interaction analyses were conducted. |
| 18 | Summarize key results with reference to study objectives. | Discussion, first paragraph |
| 19 | Discuss limitations, considering potential sources of bias or imprecision. | Limitations |
| 20 | Give a cautious overall interpretation of results, considering objectives, limitations, and relevant evidence. | Discussion; Limitations |
| 21 | Discuss the generalizability of the study results. | Discussion; Limitations |
| 22 | Give the source of funding and the role of funders. | Study Funding |
