# Supplementary material for: Employment quality and workers’ health and well-being in China: longitudinal evidence from the China Family Panel Studies
Source: J Occup Health. 2026 Aug 13;68(1):uiag046. doi: 10.1093/joccuh/uiag046 (PMC13540727; doi:10.1093/joccuh/uiag046)
Supplement: Supplementary file 1 [file supplementary_materials_uiag046.zip › Supplementary_Table_S1.docx]

**Table S1. Sample Selection Procedure**

| **Sample selection step** | **2018** | **2020** | **2022** | **Total** |
| --- | --- | --- | --- | --- |
| Original pooled sample from the three survey waves | 32,669 | 28,530 | 27,001 | 88,200 |
| Common sample with valid information on the four employment quality dimensions | 9,392 | 8,440 | 8,480 | 26,312 |
| Sample with complete control variables | 9,367 | 7,893 | 8,011 | 25,271 |
| Final sample for the self-rated health model | 9,366 | 7,826 | 7,948 | 25,140 |
| Final sample for the mental health model | 9,367 | 7,821 | 7,940 | 25,128 |
| Final sample for the happiness model | 9,362 | 7,854 | 7,966 | 25,182 |
| Final sample for the confidence in the future model | 9,365 | 7,838 | 7,955 | 25,158 |

Note: The common sample with valid information on the four employment quality dimensions required valid observations for reward, job satisfaction, working-time quality, and social security. The sample with complete control variables further required non-missing information on age, educational attainment, marital status, and rural household registration status. The final sample for each model was determined by the availability of the corresponding outcome variable.
